# AAIC draws nearly 12,000 researchers and health care professionals from around the globe to advance Alzheimer's and dementia science

**DOI:** 10.1002/alz.70853

**Published:** 2025-10-30

Researchers and health care professionals from across the globe convened in Toronto in July to learn the latest in Alzheimer's and dementia diagnosis, treatment, and care at the 2025 Alzheimer's Association International Conference^®^ (AAIC^®^).

With more than 8000 attendees joining in person and more than 3000 joining remotely, AAIC offered a choice of 148 scientific sessions, 737 podium presentations, and more than 5000 poster presentations from which attendees could advance their knowledge.

Participants represented 139 countries, and 41% were first‐time AAIC attendees. Topics included the biological underpinnings of disease, learnings in recruitment and care science, diversification of the clinical trial pipeline, treatment updates, advances in tools for detection and diagnosis, and risk factors across the lifespan. In addition, on the last day of the conference more than 8000 individuals participated in AAIC For All, a no‐cost hybrid event providing key takeaways from the conference to both the general public and health care professionals.

Following are some of the key stories reported at AAIC 2025.

## Lifestyle interventions

### U.S. POINTER

Research suggests that maintaining overall physical health may also promote brain health. Lifestyle interventions, including physical activity and eating a healthy diet, have been shown to lower dementia risk by reducing the risk of cardiovascular disease and other health factors. These connections between a healthy lifestyle and a healthy brain were confirmed by pioneering study results reported at AAIC 2025.

The Alzheimer's Association U.S. Study to Protect Brain Health Through Lifestyle Intervention to Reduce Risk (U.S. POINTER) – a 2‐year, multisite clinical trial testing two different lifestyle interventions in a diverse population of older adults at risk for cognitive decline and dementia – found that both interventions improved cognition in older adults at risk of cognitive decline.^1^ The interventions focused on physical exercise, nutrition, cognitive challenge, and social engagement, as well as heart‐health monitoring, but they differed in intensity, structure, accountability, and support provided. The more rigorous intervention, called the structured lifestyle (STR) intervention, included aerobic exercise and adherence to the Mediterranean‐DASH Intervention for Neurodegenerative Delay (MIND) diet (a diet featuring foods rich in antioxidants, omega‐3 fatty acids, and other nutrients linked to brain health). In the self‐guided lifestyle (SG) intervention, participants were allowed to select lifestyle changes that best fit their needs and schedules. While both interventions improved cognition, individuals in the STR group showed greater improvement on global cognition than those in the SG group. Specifically, the STR intervention protected cognition from normal age‐related decline for up to 2 years. This means that, relative to the SG group, participants in the STR group performed at a level comparable to adults 1 to 2 years younger – an effect that likely increases resilience against cognitive decline.

Of the 2111 participants in U.S. POINTER, more than 30% were from groups that have been historically underrepresented in dementia research. This study is the first large‐scale, randomized controlled clinical trial to demonstrate that an accessible and sustainable healthy lifestyle intervention can protect cognitive function in diverse populations in communities across the United States. Such results could lead to safe, cost‐effective, multipronged interventions for preventing or delaying dementia.

“As the burden of dementia grows worldwide, U.S. POINTER affirms a vital public health message: Healthy behavior has a powerful impact on brain health,” said Joanne Pike, Dr.P.H., Alzheimer's Association president and chief executive officer.

“The potential to improve cognition with fewer resources and lower participant burden is compelling. It highlights that while not everyone has the same access or ability to adhere to more intensive behavior interventions, even modest changes may protect the brain,” said Laura D. Baker, Ph.D., professor of gerontology and geriatrics and of internal medicine, at Wake Forest University School of Medicine and Advocate Health, and U.S. POINTER principal investigator.

The Alzheimer's Association has invested nearly $50 million to lead U.S. POINTER, with additional support from the National Institute on Aging at the National Institutes of Health for four ancillary studies. The Association will invest more than $40 million over the next 4 years to continue to follow U.S. POINTER participants, and to bring U.S. POINTER interventions to communities across America. This work will seek to understand the longer‐term impact of these lifestyle interventions, as well as collaborations with health systems and health agencies to turn the trial findings into action for people in all communities.

In addition, the Association will build on the momentum of U.S. POINTER by launching several programs and initiatives, including:

1. A personal brain health assessment tool.
2. A virtual brain health training program for health care providers.
3. A community recognition program for organizations championing brain health.
4. A brain health roundtable that will unite leaders across health care, public health, community, and corporate sectors to accelerate impact.

### Walking and brain health

In other lifestyle‐related research discussed at AAIC 2025, scientists from three international studies found that people with a higher genetic risk for Alzheimer's disease may benefit the most from healthy lifestyle interventions like walking. Older adults who carry the Alzheimer's risk gene known as APOE ε4 had higher cognitive benefits from non‐drug interventions like exercise, diet, and cognitive training than non‐carriers. Walking was found to be the most effective healthy habit for slowing cognitive decline. Like many healthy lifestyle changes, the key was making it a habit, as the study showed that sticking with the program for at least 2 years produced cognitive benefits up to 7 years later.

One of these studies included nearly 3000 older participants (mean age, 74). It found that when people increased their daily walking by 10%, they had significant improvement in overall cognitive performance. The study showed improvements of 8.5% in women and 12% in men. The researchers noted that these associations may be the result of improved delivery of oxygen and nutrients to the brain during walking – improvements that may promote the development and function of nerve cells.

### Maintaining healthy diets in low‐income communities: the SNAP study

People in low‐income communities may be at higher risk of dementia, in part because of a lack of access to healthy food. By addressing this issue, low‐income individuals may reduce dementia risk. According to new research reported at AAIC 2025, people who participated in the U.S. Supplemental Nutrition Assistance Program (SNAP) had slower cognitive decline over 10 years than non‐participants. Scientists examined data from the nationally representative Health and Retirement Study to compare participants in SNAP, which helps low‐income individuals and families buy food, with those who were eligible for the program but did not participate.

Researchers found that SNAP participants had a 0.10% slower decline in overall cognitive function. The difference is significant in the long term, adding up to an estimated 2 to 3 additional years of cognitive health over the study's 10‐year period. The study group of 1131 SNAP participants included White, Black, and Hispanic individuals. A control group included 1216 people who were SNAP‐eligible but did not participate. Researchers found that all SNAP participants benefited, but White participants showed slower decline than other groups.

The findings highlight the potential benefits of food assistance programs to support older adults’ cognitive health. They also underscore the need for public health policies that ensure equitable access to programs like SNAP, particularly for those who may face additional barriers to enrollment.

“Research has shown that food insecurity can negatively impact cognitive function, and this is one of the first long‐term studies to show that food assistance programs can positively impact cognition,” said María C. Carrillo, Ph.D., Alzheimer's Association chief science officer and medical affairs lead. “Simple, everyday actions can make a difference in brain health and may even lower the risk of Alzheimer's disease and dementia. The Alzheimer's Association is committed to helping all people build these habits into their daily lives.”

## Treatments

Many exciting clinical trial and treatment updates were reported at AAIC. Selected highlights include the following updates.

### Real‐world results for new Alzheimer's drugs show effectiveness, patient satisfaction

While newly available anti‐amyloid Alzheimer's disease drugs have shown effectiveness in tightly controlled clinical trials, they have not been tested in real‐world settings until now. Dozens of abstracts reported at AAIC 2025 showed that real‐world experience with the drugs lecanemab and donanemab produced safety comparable to or better than large clinical trials, and patients were satisfied with the results.

Researchers tracked the drugs’ safety and effectiveness in patients from a variety of settings, including U.S. clinics, memory care centers, and international universities. Several of the sites are part of the Alzheimer's Network for Treatment and Diagnostics (ALZ‐NET), which was created by the Alzheimer's Association to collect voluntary real‐world data about patients receiving the new treatments, track their long‐term health, and share data with scientists and clinicians.

### Repurposed heart‐health drugs

Scientists are also exploring whether drugs approved for other health conditions may be repurposed to protect brain health. As reported at AAIC 2025, taking a combination of common drugs used to treat blood pressure, cholesterol, and diabetes may have an added benefit: slower cognitive decline. The study examined the medical records of more than 4500 older adults from several large studies of aging. These records monitored how the individuals’ cognitive health changed over time, and they included examinations of participants’ brains at autopsy. The results showed that individuals who took a combination of drugs targeting vascular or metabolic conditions (all of which are known risk factors for dementia) had cognitive test scores similar to people 3 years younger.

Participants who were on all three of the vascular drugs enjoyed the greatest cognitive benefits, and their brains showed fewer signs of Alzheimer's‐related changes at autopsy. For those who took only two of the drugs, the most effective pairing for cognitive protection was blood pressure and cholesterol drugs.

## Risk factors

### Lead exposure

Though age is the greatest risk factor for Alzheimer's and other dementias, researchers are studying an array of other factors that may play an important role in disease risk. These factors include exposure to lead and other environmental hazards. Two studies reported at AAIC 2025 examined the effects of lead exposure on cognitive health over time. In one of them, a first‐of‐its‐kind study, researchers examined how exposure to airborne lead from 1960 to 1974 – when leaded gasoline use was at its highest – may affect brain health later in life. They determined that older adults who grew up in areas with moderate to extremely high historical atmospheric lead levels (HALL) were about 20% more likely to report memory problems as adults 50 years later than older adults who did not grow up in areas with moderate to extremely high HALL.

The researchers calculated the average HALL by area and linked it to self‐reported memory problems from the American Community Survey from 2012 to 2017 (368,208 people) and 2018 to 2021 (276,476 people). Factoring in both time periods, the researchers determined that 17% to 22% of people living in areas with moderate, high, or extremely high atmospheric lead reported memory issues.

“Our study may help us understand the pathways that contribute to some people developing dementia and Alzheimer's disease,” said Eric Brown, M.D., M.Sc., FRCPC, lead author of the study, associate scientist and associate chief of geriatric psychiatry at the Centre for Addiction and Mental Health, Toronto.

The second lead‐related study found that older adults who live about 5 km (just over 3 miles) from a lead‐releasing facility – such as glass, ready‐mixed concrete, or computer and electronics manufacturers – are more likely to have memory and cognitive problems than those who live farther away. This research, which focused on a racially and ethnically diverse group of older adults, reinforces concerns about the long‐term cognitive impact of environmental lead exposure, especially in communities already facing health disparities.

Researchers assessed 2379 older individuals from two studies: the Kaiser Healthy Aging and Diverse Life Experiences study (KHANDLE, 1638 patients) and the Study of Healthy Aging in African Americans (STAR, 741 patients). They evaluated the participants’ proximity to the nearest lead‐releasing facility and compared the distance to participants’ Neuropsychological Assessment Scales results at baseline and 2 years later. Compared with 2 years earlier, the KHANDLE participants who lived within 5 km of a lead‐releasing facility scored 0.15 times lower on verbal episodic memory tests and 0.07 times lower on overall cognitive ability compared with those living farther away. Every 5 km farther a participant lived from a lead‐releasing facility was associated with 5% higher memory scores 2 years later. Among STAR group participants living within 5 km of a lead‐releasing facility, researchers observed a 0.20 times lower score on semantic memory 2 years later compared with those who lived farther away.

“Our results indicate that lead exposure in adulthood could contribute to worse cognitive performance within a few years,” said Kathryn Conlon, Ph.D., M.P.H., senior author and associate professor of environmental epidemiology, School of Medicine, University of California, Davis. “Despite tremendous progress on lead abatement, studies have shown there is no safe level of exposure, and half of U.S. children have detectable levels of lead in their blood. Additionally, there are regions and neighborhoods that have more exposure.”

### Risk factors for women

According to the *2025 Alzheimer's Disease Facts and Figures* report, the reasons men and women develop dementia may differ.^2^ Two studies reported at AAIC 2025 focused on risk factors that may have an especially profound impact on women's brain health. Women make up nearly two‐thirds of the more than seven million Americans living with Alzheimer's.

One of the studies focused on traumatic brain injury (TBI). TBIs can promote dementia in both men and women, but researchers in this study found that TBIs were more likely to shrink dementia‐related areas of the brain in women than in men.

The other study took a closer look at “chemobrain” – declines in cognition and memory reported by about one‐third of women receiving chemotherapy. This study is the first to show that brain changes, inflammation, and shrinkage related to cancer treatment are associated with symptoms such as memory lapses and trouble focusing or finding words. The study adds to growing evidence that chemotherapy impacts brain health. It also helps us better understand sex‐based differences in cognitive health.

## New diagnostic guideline: Blood‐based biomarkers in specialty settings

Techniques for diagnosing Alzheimer's disease have been undergoing a transformation in recent years. While traditional diagnostic methods have focused on cognitive testing, biomarkers in the brain, blood, and cerebrospinal fluid (CSF) may indicate disease at a much earlier stage – when treatments are most effective. Biomarkers include dementia‐related proteins (such as amyloid beta) as well as abnormal inflammation. However, many biomarker diagnostic tests, including positron emission tomography (PET) scans of the brain and lumbar punctures to examine CSF, are costly and invasive. Blood‐based biomarker (BBM) tests, on the other hand, can provide diagnostic accuracy as well as greater affordability and acceptability to patients.

At AAIC 2025, the Alzheimer's Association a landmark step toward transforming Alzheimer's disease diagnosis in specialty care. The Association released its first clinical practice guideline (CPG) on the use of BBM tests.^3^ The CPG provides clear, evidence‐based, brand‐agnostic recommendations to support more accurate and accessible diagnosis of Alzheimer's using BBM tests. Its recommendations are based on a systematic review using a robust and transparent methodology and will be updated regularly as evidence evolves.

The CPG is designed for specialists involved in the diagnostic evaluation of cognitive impairment in specialized care settings. A specialist is defined as a health care provider, typically in neurology, psychiatry, or geriatrics, who cares for adults with cognitive impairment or dementia. It also applies to primary care providers, nurse practitioners, and physician assistants in specialized care settings.

To determine the guidelines, a panel of 11 clinicians convened by the Alzheimer's Association – including clinical neurologists, geriatricians, nurse practitioners, physician assistants, and subject‐matter experts – conducted a systematic review of various name‐brand BBM tests. These tests involved, for example, blood‐based tau and amyloid beta proteins. In the end, the panel determined that endorsing specific tests was premature, opting for a brand‐agnostic approach that blinded panel members to the tests they were evaluating to minimize bias. This ensures the guideline's credibility, durability, and actionability. According to the panel: “Ranking or endorsing specific tests is premature at this time. Instead, test accuracy data and accuracy judgments reported in this guideline are meant to serve as a resource for clinicians … to aid them in choosing which test(s) to order.”

The panel formulated two recommendations and one Good Practice Statement for using BBM tests in the diagnostic workup of individuals with objective cognitive impairment being seen in specialized care. The recommendations suggest the use of different quality BBM tests as (1) a preliminary assessment or (2) a confirmatory test in the diagnostic workup of Alzheimer's disease. The Good Practice Statement urges that a BBM test should not be obtained before a comprehensive clinical evaluation by a health care professional and that test results should always be interpreted within the clinical context. Moreover, clinicians should consider the pretest probability of Alzheimer's disease pathology each patient when deciding whether to use a BBM test.

This CPG is part of ALZPro, the Alzheimer's Association's comprehensive hub of resources to promote best practices, empowering health professionals across disciplines to reduce risk, advance early detection, improve care, and expand equitable access for all communities. ALZPro unites care resources, relevant scientific findings, clinical guidelines and insights, continuing education, and implementation tools on one platform.

“This is a pivotal moment in Alzheimer's care,” said Carrillo, a co‐author of the guideline. “For the first time, we have a rigorously evidence‐based guideline that empowers clinicians to use blood biomarker tests confidently and consistently. Adoption of these recommendations will lead to quicker, more accessible, more accurate diagnoses – and better outcomes for individuals and families affected by Alzheimer's.”

## Awards

Ten awards were presented at AAIC 2025 recognizing researchers for their expertise, noteworthy achievements, and innovative contributions to the field of Alzheimer's disease and dementia science.

“The Alzheimer's Association envisions a world without Alzheimer's and all other dementia, and these leaders are helping us make that vision a reality,” said Carrillo. “The scientific leaders and innovators we recognize at AAIC 2025 are exploring new frontiers in research, medicine, and care. We thank them for their impressive accomplishments and insights, and their dedication to the cause.”

### Bill Thies Award

The Bill Thies Award for Distinguished Service to the International Society to Advance Alzheimer's Research and Treatment (ISTAART) recognizes a society member who has provided continued and outstanding service and mentorship to the ISTAART community. Donna M. Wilcock, Ph.D., is the recipient of this year's Bill Thies Award. She is professor of neurology and director of the Center for Neurodegenerative Disorders at Indiana University (IU) School of Medicine and IU Health. She is also the Barbara and Larry Sharp Professor in Alzheimer's Disease Research and a member of Stark Neurosciences Research Institute and leads the Biomarker Core for the Indiana Alzheimer's Disease Research Center. Wilcock served on the ISTAART Advisory Council (IAC) from 2016 and chaired the IAC from 2020 to 2022. Her steadfast dedication to mentoring and advancing junior scientists has continued into her current role as the editor‐in‐chief of *Alzheimer's & Dementia: The Journal of the Alzheimer's Association*.

### Inge Grundke–Iqbal Award

Katrin Andreasson, M.D., is this year's recipient of the Inge Grundke–Iqbal Award for Alzheimer's Research. This award is presented to the senior author of the most impactful study published in Alzheimer's research during the two calendar years preceding AAIC. Her paper, “Restoring hippocampal glucose metabolism rescues cognition across Alzheimer's disease pathologies,” was published in the journal *Science* in August 2024^4^. Andreasson is a physician‐scientist and the Edward F. and Irene Thiele Pimley Professor of Neurology and Neurological Sciences at Stanford University School of Medicine.

### Zaven Khachaturian Award

Named in honor of noted scientist, administrator, consultant, lecturer, and author Zaven Khachaturian, Ph.D., this award recognizes an individual whose compelling vision, selfless dedication, and extraordinary achievement has significantly advanced the field of Alzheimer's science. Bruce Lamb, Ph.D., is the 2025 recipient of the award. He is an Indiana University Distinguished Professor, Roberts Family Chair in Alzheimer's Disease Research, executive director of the Stark Neurosciences Research Institute at the Indiana University School of Medicine, and co‐director of the Neuroscience Institute at Indiana University Health. Dr. Lamb is a member of the Alzheimer's Association Board of Directors, immediate‐past chair of the Association's Medical and Scientific Advisory Group and life‐long volunteer and advocate for Alzheimer's and dementia research.

### De Leon Prizes in Neuroimaging

The de Leon Prizes in Neuroimaging recognize scientists from ISTAART's community of researchers and clinicians judged to have published “best papers” in the field of neuroimaging of neurodegenerative processes. The awards are named after Mony J. de Leon, Ed.D., professor of psychiatry and director, Center for Brain Health at New York University Langone Health, and one of the founders of the Alzheimer's Imaging Consortium. The 2025 de Leon Prize honorees are:

1. Senior Scientist: Christos Davatzikos, Ph.D., University of Pennsylvania, United States
2. Junior Scientists: Hironobu Endo, M.D., Ph.D., and Maiko Ono, Ph.D., both of the National Institutes for Quantum Science and Technology, Japan
3. Trainee: Karly Cody, Ph.D., Stanford University, United States

### AAIC Lifetime Achievement Awards

The AAIC Lifetime Achievement Awards are named in honor of Henry Wisniewski, M.D., Ph.D.; Khalid Iqbal, Ph.D.; and Bengt Winblad, M.D., Ph.D.—co‐founders of the International Conference on Alzheimer's Disease, now known as the Alzheimer's Association International Conference. These awards honor significant contributions to Alzheimer's and dementia research, either through a single scientific discovery or a body of work.

Bart de Strooper, M.D., Ph.D., is the recipient of the 2025 Khalid Iqbal Lifetime Achievement Award. He is a professor in dementia research at KU Leuven in Belgium and the founding director of the United Kingdom Dementia Research Institute. De Strooper is internationally recognized for his pioneering research into the mechanisms underlying Alzheimer's disease. De Strooper's more recent research has focused on how amyloid plaques provoke a pathological multicellular neuroinflammatory response, with immune cells called microglia and astrocytes as key drivers.

Clifford R. Jack Jr., M.D., is the recipient of the 2025 Henry Wisniewski Lifetime Achievement Award. Jack is professor of radiology, specializing in neuroradiology, and the Alexander Family Professor of Alzheimer's Disease Research at Mayo Clinic in Rochester, Minnesota, in the United States. As a global expert in this space, his Aging and Dementia Imaging Research lab is engaged in brain imaging research in cognitive aging and Alzheimer's disease and related disorders. Jack is world renowned for criteria and frameworks that are fundamental to our current understanding of neurodegenerative disease.

Mary Sano, Ph.D., is the recipient of the 2025 Bengt Winblad Lifetime Achievement Award. Sano is a professor of psychiatry and the director of the Alzheimer's Disease Research Center at the Icahn School of Medicine at Mount Sinai in New York City. Sano is a neuropsychologist by training and an award‐winning clinical trialist, focusing on cognitive impairment in aging and Alzheimer's disease and related dementias. Her work has focused on understanding the cognitive consequences of disease and discovery of interventions to improve cognition.

For more information about these award recipients and the discoveries presented at AAIC, visit https://aaic.alz.org/. Mark your calendars for AAIC 2026, to be held July 12 to 16 in London, UK, and online.
